# Genomic variation in *Salmonella enterica *core genes for epidemiological typing

**DOI:** 10.1186/1471-2164-13-88

**Published:** 2012-03-12

**Authors:** Pimlapas Leekitcharoenphon, Oksana Lukjancenko, Carsten Friis, Frank M Aarestrup, David W Ussery

**Affiliations:** 1 National Food Institute, Building 204, The Technical University of Denmark, 2800 Kgs Lyngby, Denmark; 2 Center for Biological Sequence Analysis, Department of Systems Biology, Technical University of Denmark, Building 208, DK-2800 Kgs Lyngby, Denmark

## Abstract

**Background:**

Technological advances in high throughput genome sequencing are making whole genome sequencing (WGS) available as a routine tool for bacterial typing. Standardized procedures for identification of relevant genes and of variation are needed to enable comparison between studies and over time. The core genes--the genes that are conserved in all (or most) members of a genus or species--are potentially good candidates for investigating genomic variation in phylogeny and epidemiology.

**Results:**

We identify a set of 2,882 core genes clusters based on 73 publicly available *Salmonella enterica *genomes and evaluate their value as typing targets, comparing whole genome typing and traditional methods such as 16S and MLST. A consensus tree based on variation of core genes gives much better resolution than 16S and MLST; the pan-genome family tree is similar to the consensus tree, but with higher confidence. The core genes can be divided into two categories: a few highly variable genes and a larger set of conserved core genes, with low variance. For the most variable core genes, the variance in amino acid sequences is higher than for the corresponding nucleotide sequences, suggesting that there is a positive selection towards mutations leading to amino acid changes.

**Conclusions:**

Genomic variation within the core genome is useful for investigating molecular evolution and providing candidate genes for bacterial genome typing. Identification of genes with different degrees of variation is important especially in trend analysis.

## Background

With the increasing number of available bacterial genome sequences, when these genomes are compared, the genetic variation within bacterial species is greater than previously predicted [1,2]. Rapid and reliable sub-typing of bacterial pathogens is important for identification of outbreaks and monitoring of trends in order to establish population structure and to study the evolution among bacterial genomes especially within and between the outbreak strains. Today, the most widely used typing methods for bacterial genomes include multilocus sequence typing (MLST), pulsed field gel electrophoresis (PFGE), sequencing of 16S rRNA genes, and multilocus variable-number of tandem-repeat analysis (MLVA).

PFGE and MLVA have major benefits, but are time consuming and the results are difficult to standardize [3]. Other typing methods which rely on one or a few ubiquitous genes, such as the 16S rRNA gene or a set of housekeeping genes in MLST, are capable of classification at the species level and sometimes also at the subspecies level, but the biological information in a narrow selection of genes will rarely be sufficient to clearly distinguish between closely related strains such as several isolates of the same serotype [4-6]. Thus, more of the genome content should be considered rather than just one or a few genes [4].

The price and time for whole genome sequencing will soon be in the same range as the traditional typing methods mentioned above. Genome sequencing can be a powerful method in epidemiological and evolutionary investigations [7-9]. Although, to date, this has only been used in more limited epidemiological investigations where isolates suspected to be part of the same outbreak have been compared to a reference genome. In the future, it is likely that WGS will become a routine tool for identification and characterization of bacterial isolates, as hinted at in the first 'real-time' sequencing of the *E. coli *O104 outbreak in Germany in the summer of 2011 [10] and the *Vibrio cholerae *outbreak in Haiti in October 2010 [11]. This requires standard procedures for identifying variation and for analyzing similarities and differences.

Conserved genes are present across bacterial genomes of the same species (or genus). A fraction of these genes--those conserved in all (or most) of the genomes of a given bacterial taxonomic group--is called the 'core-genome' of that group. The core-genome can be identified either within a genus or species [3] and can be used to identify the variable genes in a given genome [12]. In addition, the conserved genes in general appear to evolve more slowly, and can be used for determining relationships among bacterial isolates [13].

Currently there are more than a hundred bacterial species for which sufficient genomic data are available to estimate the species core-genome (that is, there are at least three genomes sequenced from the same species) [14]. Among these, *Salmonella enterica *is a good candidate species for conserved gene identification because the genomes are quite similar [15]. Moreover, *S. enterica *is one of the most important food-borne pathogens and is responsible for global outbreaks [16] which makes international standard typing procedures of major importance in order to allow for global comparisons [17]. The *Salmonella *genus has only two species with sequenced genomes: *Salmonella bongori *and *Salmonella enterica*. In turn, *S. enterica *is divided into 6 sub-species: *enterica, salamae, arizonae, diarizone, houtenae *and *indica*. Presently, *S. enterica *is classified into more than 2,500 serotypes [18].

In order to investigate an outbreak caused by *Salmonella*, characterization of *Salmonella *isolates from genome data is a crucial step. *Salmonella *genomes are highly similar, particularly within subspecies *enterica*, where little variance exists in the genomes [15]. This high similarity presents a challenge for typing and classification.

In their pioneering work Tettelin *et al. *[1] defined the core genes of a species by being those genes found present in (nearly) all known members of the species. Since then others have studied core and pan genomes at the genus level or even at the kingdom level [19], but for our purposes the original definition at the species level is suitable. In this work we identify the core genes within *S. enterica *genomes and determine variation between the different available genomes, both in terms of sequence and presence/absence of non-core genes; in the latter case using a method originally published by Snipen & Ussery [20]. We evaluate the value of different approaches for classification of isolates in epidemiological settings and compare our findings to currently used sequencing methods, both in long term trend analysis and outbreak investigations.

## Results and discussion

The 73 *Salmonella *genomes used in this study are summarized in Additional file 1: Table S1. The set comprises 21 completed genomes and 52 nearly completed genomes. Of these, 35 genomes are closely-related *S*. Montevideo strains pertaining to an outbreak of salmonellosis from Italian-style spiced meat [21]. All genomes were retrived from GenBank [22] except *S*. Typhimurium str. DT104, which was received from the Sanger Institute's bacterial genome database. All *Salmonella *genomes are from subspecies enterica with the exception of the single *S. enterica subsp. Arizonae*.

### Evaluation of traditional bacterial sequence-based typing

The ribosomal genes are essential for the survival of all cells, and their structure cannot change much because of their involvement in protein synthesis [23]. Thus, 16S rRNA genes are highly conserved among isolates belonging to the same bacterial species [4]. Exceptions may be *N. meningitidis *[24] and *Mycoplasma *[25]. However, due to limited variation within a given species, the 16S sequencing is often not useful for epidemiological studies, where the classification of highly similar strains is needed. Jacobsen *et al. *shows a phylogenetic tree based on 16S rRNA genes, extracted from 26 *Salmonella enterica *genomes, using RNAmmer [15,26]. As expected, there is not sufficient resolution to distinguish among the *Salmonella *subspecies *enterica*.

Genes such as *rpoB *or *sodA *have been suggested as substitutes for 16S rRNA and have shown improved efficacy in species identification [27], although it remains unlikely that a single gene can always reflect the subtle differences between genomes of the same species.

**Figure 1:**
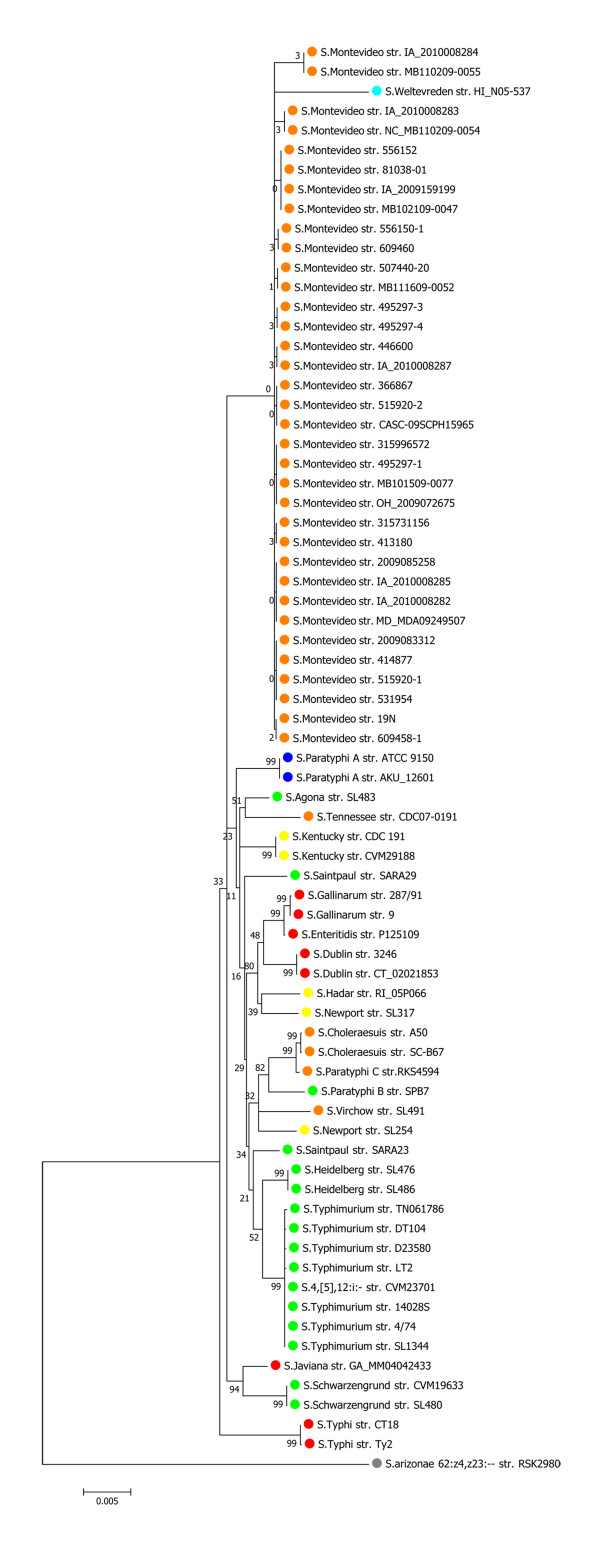
***In silico *MLST tree**. Seven housekeeping genes were extracted from *Salmonella *genomes. Concatenated sequences were aligned by MUSCLE. The phylogenetic trees were generated by MEGA5 using bootstrap maximum likelihood method. Each color represents a different serogroup (O antigen). The confidence value is the bootstrap value calculated by sampling with replacement from the multiple sequence alignment.

The limitations of using a single gene may be improved by the simultaneous analysis of multiple genes. Multi Locus Sequence Typing (MLST) has found wide applications, especially in phylogenetic studies and is most commonly based on seven housekeeping genes - each bacterial species having its own set. For *Salmonella *these are: *aroC, dnaN, hemD, hisD, purE, sucA *and *thrA *http://www.mlst.net. A MLST tree, based on an *in silico *analysis of the 73 available *Salmonella enterica *genomes in Genbank, is shown in Figure 1. Strains of the same serovar generally cluster into distinct groups, although exceptions exist; for example the *S*. Weltevreden str. HI_N05-537 is mixed with *S*. Montivideo. Futhermore, recent work on 61 sequenced *E. coli *genomes [4], found that the 16S rRNA tree cannot resolve well within the genus level and also that MLST cannot differentiate pathogenic strains from non- pathogenic strains. Still, MLST has proven useful for long-term analysis of population structures, but often fails to detect differences between closely related strains [28]. Indeed, improved MLST schemes that include more than 7 genes have been suggested [4].

For *Salmonella*, sequencing specific short repeats and virulence genes have recently been suggested as an alternative and improved method for typing of *S. Enteritidis*[29]. The usefulness of this approach in epidemiological studies and typing is currently unknown, although the choice of repeats must be tailored for the specific bacterial species studies.

### Identification of core genes

**Figure 2:**
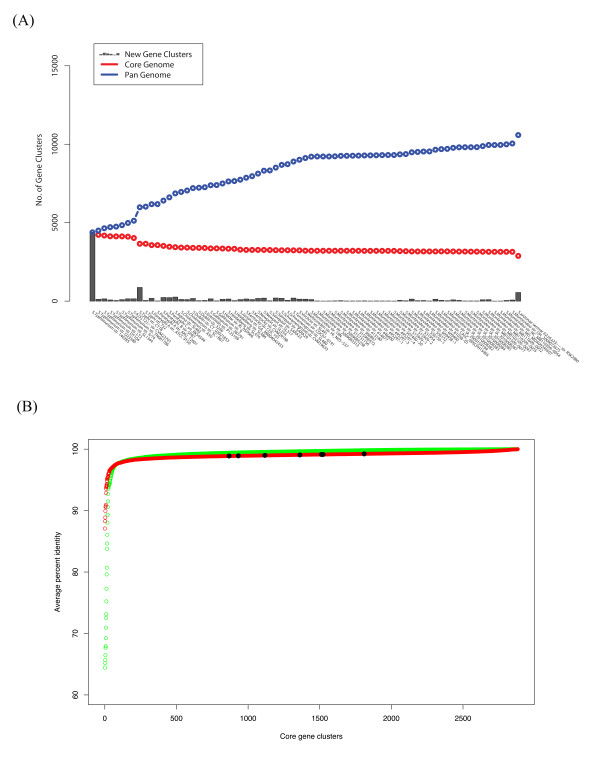
**Pan- core-genome plot and variation plot. (A)** Pan- and core-genome plot of 73 *Salmonella enterica*. The plot shows an increase of the pan-genome (blue line) and a decrease of the core-genome (red line) as more genomes are added. The last points show the total number of gene clusters in the pan-genome and the core-genome. **(B)** Variation plot. This plot shows the variation within core gene clusters in amino acid levels (green dots) and nucleotide levels (red dots). Black dots show the distribution of housekeeping genes in the core genes. The Y- and X-axes represent average percent identity and numerical core gene cluster name respectively.

Determining gene conservation across multiple genomes is not overly difficult, but certain choices must be made which will affect the final outcome. Using a previously published method [20,30,31] which employs single-linkage clustering on top of BLASTp alignments, sets of pan- and core-genomes were estimated, based on all 73 *Salmonella *genomes. The progression of the pan- and core-genomes is shown in Figure 2A. The number of novel gene clusters in the pan-genome gradually increases when more genomes are considered, while the number of conserved gene clusters constituting the core genome decreases slightly. When all *Salmonella *genomes have been considered, there are 10,581 pan gene clusters and 2,882 core gene clusters (Additional file 2) in species *enterica*. In the step going from *S*. Typhimurium to *S*. Typhi, the number of core genes drops suddenly, most likely because the *S*. Typhi genome has undergone considerable pseudogene formation resulting in gene loss [32]. The number of core genes drops again when adding a genome of the subspecies *arizonae *which is associated with cold-blooded animals. This technique has previously been applied successfully in finding core genomes for Proteobacteria genera *Burkholderia *[33], *Escherichia coli *[4], *Vibrionaceae *[34] and *Campylobacter jenuni *[30], as well as Bacteroides [35] and Lactic acid bacteria [36].

### Genomic variation within the core genes

The core genes as calculated above were used for constructing a gene variation plot by performing all-against-all BLAST alignments between 2,882 core gene clusters and all 73 *Salmonella enterica *genomes. The resulting average identities within each core gene cluster is displayed in Figure 2B. From this figure, the average percent identity was very high (> 98%) in most of the core genes, but dropped sharply for around 5% of the core genes. From this plot, the identified core genes can be divided into two categories: a small group of highly variable genes and the majority of genes which show little variation.

For the highly variable core genes, the variation in amino acid sequences (Figure 2B, green dots) was higher than for the nucleotide sequences (Figure 2B, red dots), whereas the opposite was the case for the more conserved core genes. This indicates that for core genes with low variation there is a selection against mutations leading to amino acid changes, whereas for the highly variable genes, positive selection for amino acid changes seems to be the case. In order to confirm these hypothesis, the approximation of dN/dS has been performed by dividing the number of non-synonymous changes per non-synonymous sites with the number of synnonymous changes per synonymous sites [37] using *S*. Typhimurium str. LT2 as a reference genome. The median dN/dS ratio for conserved and highly variable core genes are 1.0 and 1.25 respectively. Therefore, the amino acid changes in highly variable core genes might be due to an increase in positive selection at some sites. Nonetheless, the importance of this needs to be confirmed by additional analysis, although one could imagine, for example, a selective pressure to vary the surface proteins to avoid immune response.

The seven genes used for MLST are marked in the Figure 2B, and are scattered throughout the highly conserved part of the core genes (Figure 2B, black dots) and, as expected, little variation exists in these genes. Including core genes from both the highly conserved and variable regions might be beneficial in evolution studies. On the one hand, the more slowly evolving genes are useful in distinguishing between divergent and convergent evolution, while faster evolving genes can help in strain identification.

### Functional analysis of conserved genes

**Figure 3:**
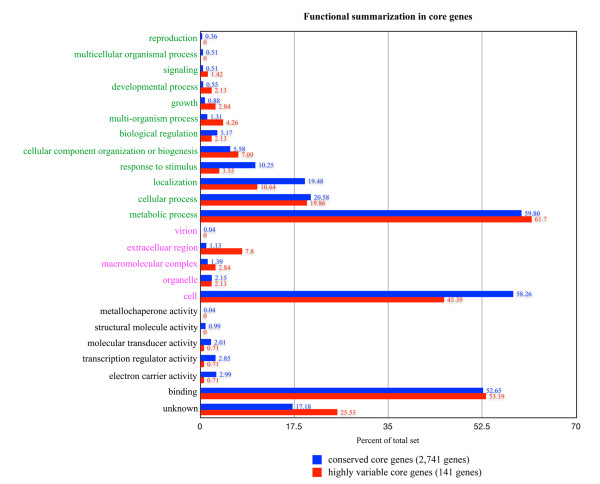
**Gene Ontology term summary of core genes**. Gene Ontology terms for conserved core genes (blue bars) and highly variable core genes (red bars) are shown in 3 categories (from top to bottom): biological processes (green labels), cellular component (pink labels) and molecular function (black labels). GO are assigned from blast all-against-all between core genes and protein sequences from Uniprot based on 50/50 rule. All conclusions drawn about the variable set are relative to the fraction of like sequences in the conserved set, and not in any way absolute.

In order to determine the functional profile of core genes, the core gene clusters were aligned against UniProt [30]. Functional profiles were determined based on Gene Ontology (GO) terms and visualized in Figure 3. Though the difference is generally small, some terms common in conserved core genes tend to be less frequent in highly variable core genes; for example, electron carrier activity, structural molecule activity and metallochaperone activity. These functions are essential for living cells and are therefore enriched in conserved core genes. On the other hand, highly variable core genes encode many proteins that are associated with the extracellular region. In general, genes located outside the cell are known to be more variable [38].

### Consensus tree based on core gene clusters

**Figure 4:**
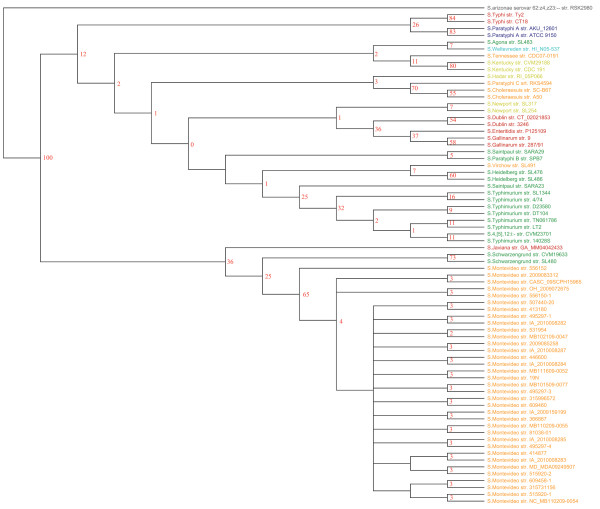
**Consensus tree based on 2,882 core gene clusters**. Phylogenetic trees were constructed from all core genes using PAUP. All trees were combined and the consensus trees were generated using the Phylip software package. The percentage of branches present in all trees is shown. The colors represent different serogroups, as in Figure 1.

Figure 4 shows a phylogenetic tree generated from the sequence of all 2,882 *Salmonella *core gene clusters. The tree generally divides the serotypes up well, but the bootstrap value in several branches is very low. This uncertainty could be due to the large number of core gene trees being analyzed individually; the low bootstrap values near the root reflect a lack of consensus at the higher levels. In contrast, the low bootstrap values found in *S*. Montevideo strains likely reflect uncertainty due to the high similarity of gene sequence of the clonal outbreak. All *S*. Montevideo strains sequenced were from a single outbreak [21] and as expected this analysis confirmed the almost complete identity of these isolates.

A previous study described that there are 69 genes unique to *Salmonella *[39]. Instead of using all core genes, we generated a consensus tree based on these 69 *Salmonella-*specific genes (Additional file 3: Figure S1). We also constructed an additional four consensus trees based on sets of 69 core genes randomly picked from different areas in the variation plot (Figure 2B): from a mixture of high, medium and low variable core genes (Additional file 4: Figure S2), from medium variable core genes (Additional file 5: Figure S3), from highly variable core genes (Additional file 6: Figure S4) and from the area where the curve decreases in the variation plot (Additional file 7: Figure S5). The appearance of these 5 consensus trees was similar to the tree from Figure 4, with two exceptions: the trees based on the 69 specific genes (Additional file 3: Figure S1) and the highly variable core genes (Additional file 6: Figure S4). In the former, *S. arizonae*, which is not part of the subspecies *enterica*, was still mixed in with other *enterica*, while for the latter, *S*. Agona str. SL483 clustered away from the other subspecies *enterica*. Thus, based on these results, it appears that using only *Salmonella *unique genes or highly variable genes does not provide phylogenetically useful information and should probably not be used for future WGS studies. Comparisons using more genomes in more species can further test this.

### Pan-genome tree

**Figure 5:**
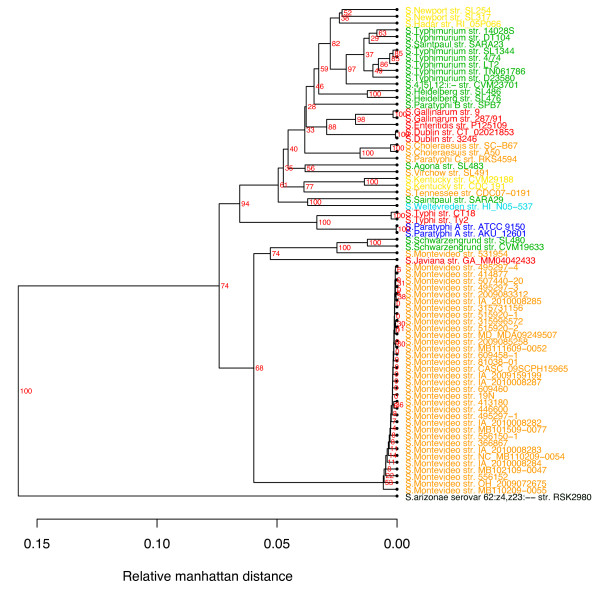
**Pan-genome tree**. This tree does not produce a sequence-based alignment tree but it is generated from the presence or absence of gene clusters across all *Salmonella *genomes [31]. The bootstrap values are shown in red.

In principle, genome similarity is not only measurable by shared genes, but also by the absence of genes. Figure 5 is another tree, based on gene presence/absence across all the *Salmonella *genomes [20]. This tree bears a striking resemblance to the consensus tree based on core genes (Figure 4), although the bootstrap values are higher in many of the branches, especially near the root. Of all methods investigated in this study, the pan-genome tree presents itself as the best solution for a tree that can resolve strain differences in a biologically meaningful way, even if it would be expected to correlate more with phenotype than phylogeny. It is, however, important to note that creating pan-genome trees requires higher quality sequencing data and assemblies than what are typically obtained using short reads from second-generation sequencing methodologies. Even so, we have found that pan-genome trees with good correspondence to known bacterial types can be constructed from Solexa data (100 bp reads), if care is taken to ensure good assembly and gene finding (data not shown).

The power to discriminate between variants differs between the methods used. The phylogenetic analysis for the MLST tree is based on the identified informative sites among the seven housekeeping genes, for the pan-genome tree on presence and absence of genes and for the consensus tree based on the informative sites of core gene clusters from alignments of all core genes trees. The number of infomative sites for *in silico *MLST tree, pan-genome tree and consensus tree based on core gene clusters were 877 bp (10,008 total base-pairs in the seven genes), 7,699 genes (10,581 total genes) and 880,832 bp (2,868,821 bp in all core genes), respectively. The pan genome and core gene analysis were based on much more variation than the MLST analysis and have a much stronger power to discriminate closely related strains.

## Conclusions

Bacterial typing should provide meaningful information for both epidemiological and evolutionary studies. For epidemiology, the ability to differentiate unrelated isolates (discriminatory power) and the ability to cluster related isolates are crucial. 16S rRNA and the MLST genes rarely provide separation between closely related strains. The performance of the pan-genome tree, however, is valid for epidemiological investigation in both discriminatory and clustering abilities. One caveat is that this method depends on good quality genomic data.

Comparative genomics can determine the conserved genes (core-genome) among bacterial genomes at either genus or species level. Genomic variation within the core-genome can then be used to reveal highly variable genes (fast evolving genes) and conserved genes (slow evolving genes). These core genes are useful for investigating molecular evolution and remain useful as candidate genes for bacterial genome typing--even if they cannot be expected to differentiate highly similar isolates from e.g. outbreak cases, such is not always desirable. Even in cases where a deeper distinction of isolates is of interest, e.g. in mapping outbreaks, core genes might still be useful as a reference fragment for SNPs calling instead of using whole genome analysis. However, in term of computational costs, the consensus tree based on core genes requires more computational time than the other methods.

In the near future, global real-time surveillance of *Salmonella *and other pathogens giving simultaneous information on population structure and evolution, as well as outbreak detection, may well be possible.

## Methods

### *Salmonella *genome data and gene annotation

From public genome databases (NCBI and Sanger Institute's bacterial genome databases), 83 *Salmonella enterica *genomes available at the time (April, 2011) were downloaded. These genomes consisted of 21 completed genomes and 62 draft genomes. Due to the large number of contigs in some genomes, only 73 genomes were selected for this study (Additional file 1: Table 1). The gene finder Prodigal was used on DNA sequences of all genomes to eliminate biases in annotation quality and to standardize the genes found in all genomes [15]. Gene clusters were then inferred according to [15,20,30]

### *In silico *MLST trees

The *in silico *MLST tree was constructed from seven housekeeping genes: *aroC, dnaN, hemD, hisD, purE, sucA *and *thrA *http://www.mlst.net. These genes were extracted from *Salmonella *genomes and concatenated. The concatenated sequences were aligned using MUSCLE [40]. Phylogenetic trees were generated by MEGA5 using the maximum likelihood method [41]. The confidence value is, in this case, the same as the bootstrap value, calculated by sampling with replacement from the multiple sequence alignments [42]. Thus, the *in silico *MLST differs from traditional MLST in that complete genes are used and not just the MLST alleles. However, since the alleles typically cover the majority of the genes, the difference is small.

### Consensus trees

All core gene clusters from 73 *Salmonella *genomes were used for generating a consensus tree. Multiple alignments for each core gene cluster from all strains were performed using MUSCLE [40]. A phylogenetic tree for each core gene was generated using PAUP [43]. The Phylip package was used to construct the consensus tree from all the trees [44]. The bootstrap values are shown in the consensus tree.

### GO annotation

The core gene clusters were compared in an all-against-all BLAST with protein sequences from UniProt based on the '50/50 rule' [30]. Functional profiles were summarized from BLAST results by mapping UniProt IDs to Gene Ontology (GO) terms. Mapping GO parental terms were performed using publicly available GO-PERL modules for searching through a graph structure of ontology data [45,46]

### Pan-genome trees

The Pan-genome matrix consists of gene clusters (rows) and genomes (columns). The absence and presence of genes across genomes are represented by 0's and 1's respectively. The relative Manhattan distance between genomes was calculated and used for hierarchical clustering. The bootstrap values are calculated in order to represent the confidence of branches [20].

## Competing interests

The authors declare that they have no competing interests.

## Authors' contributions

PL planned the study, carried out all bioinformatics analysis and drafted the manuscript. OL participated in consensus tree based on core genes. CF participated in the planning of the study, the core genes identification and drafted the manuscript. FMA supervised and planned the study and drafted the manuscript. DWU supported the supervision, participated in the design of the study and drafted the manuscript. All authors have read and approved the final manuscript.

## Supplementary Material

Additional file 1 **Table S1 **List of *Salmonella *genomes used in this study. Click here for file

Additional file 2 **Core gene clusters**. This file contains 2,882 *Salmonella *core genes in FASTA format. Click here for file

Additional file 3 **Figure S1 **Consensus tree based on 69 specific *Salmonella *genes. Click here for file

Additional file 4 **Figure S2 **Consensus tree based on 69 *Salmonella *core genes randomly picked up from high, medium and low variable core genes. Click here for file

Additional file 5 **Figure S3 **Consensus tree based on 69 Salmonella core genes randomly picked up from medium variable core genes. Click here for file

Additional file 6 **Figure S4 **Consensus tree based on 69 Salmonella core genes randomly picked up from highly variable core genes. Click here for file

Additional file 7 **Figure S5 **Consensus tree based on 69 Salmonella core genes randomly picked up from decreasing curve in the variation plot. Click here for file
