# Supplementary material for: Genomic variation in Salmonella enterica core genes for epidemiological typing
Source: BMC Genomics. 2012 Mar 12;13:88. doi: 10.1186/1471-2164-13-88 (PMC3359268; doi:10.1186/1471-2164-13-88)
Supplement: Additional file 1 — Table S1 List of Salmonella genomes used in this study. [file 1471-2164-13-88-S1.PDF]

Table 1 List of *Salmonella* genomes used in this study.

GPID	Organism	Status	Contigs	Genes	Serogroup (O antigen)	color
**30943**	S.Paratyphi A str. AKU_12601	complete	1	4351	O:2	blue
**13086**	S.Paratyphi A str. ATCC 9150	complete	1	4348	O:2	blue
**19465**	S.4,[5],12:i:- str. CVM23701	assembly	113	4694	O:4	green
**20063**	S.Agona str. SL483	complete	2	4508	O:4	green
**20045**	S.Heidelberg str. SL476	complete	3	4680	O:4	green
**20065**	S.Heidelberg str. SL486	assembly	48	4432	O:4	green
**27803**	S.Paratyphi B str. SPB7	complete	1	4561	O:4	green
**19461**	S.Saintpaul str. SARA23	assembly	2	4350	O:4	green
**19463**	S.Saintpaul str. SARA29	assembly	182	4757	O:4	green
**19459**	S.Schwarzengrund str. CVM19633	complete	3	4550	O:4	green
**20071**	S.Schwarzengrund str. SL480	assembly	67	4547	O:4	green
**33067**	S.Typhimurium str. 14028S	complete	2	4653	O:4	green
**56087**	S.Typhimurium str. 4/74	complete	4	4771	O:4	green
**241**	S.Typhimurium str. LT2	complete	2	4635	O:4	green
**50407**	S.Typhimurium str. SL1344	complete	1	4549	O:4	green
**40625**	S.Typhimurium str. D23580	complete	1	4562	O:4	green
**61789**	S.Typhimurium str. TN061786	assembly	24	4626	O:4	green
**-**	S.Typhimurium str. DT104	complete	2	4635	O:4	green
**53347**	S.Choleraesuis str. A50	assembly	18	9130	O:7	orange
**9618**	S.Choleraesuis str. SC-B67	complete	3	4792	O:7	orange
**48457**	S.Montevideo str. 19N	assembly	60	4364	O:7	orange
**51389**	S.Montevideo str. 2009083312	assembly	30	4340	O:7	orange
**51391**	S.Montevideo str. 2009085258	assembly	34	4339	O:7	orange
**51393**	S.Montevideo str. 315731156	assembly	40	4354	O:7	orange
**46535**	S.Montevideo str. 315996572	assembly	50	4364	O:7	orange
**49989**	S.Montevideo str. 366867	assembly	37	4358	O:7	orange
**49991**	S.Montevideo str. 413180	assembly	38	4349	O:7	orange
**49987**	S.Montevideo str. 414877	assembly	46	4357	O:7	orange
**49993**	S.Montevideo str. 446600	assembly	47	4354	O:7	orange
**46539**	S.Montevideo str. 495297-1	assembly	51	4360	O:7	orange
**46541**	S.Montevideo str. 495297-3	assembly	39	4342	O:7	orange
**46543**	S.Montevideo str. 495297-4	assembly	44	4358	O:7	orange
**50023**	S.Montevideo str. 507440-20	assembly	44	4348	O:7	orange
**46545**	S.Montevideo str. 515920-1	assembly	39	4346	O:7	orange
**46547**	S.Montevideo str. 515920-2	assembly	34	4342	O:7	orange
**46549**	S.Montevideo str. 531954	assembly	132	4059	O:7	orange
**49997**	S.Montevideo str. 556150-1	assembly	83	4371	O:7	orange
**51379**	S.Montevideo str. 556152	assembly	173	4483	O:7	orange
**49995**	S.Montevideo str. 609458-1	assembly	98	4378	O:7	orange
**50021**	S.Montevideo str. 609460	assembly	95	4386	O:7	orange
**49129**	S.Montevideo str. 81038-01	assembly	39	4347	O:7	orange
**46907**	S.Montevideo str. CASC_09SCPH15965	assembly	69	4512	O:7	orange
**51975**	S.Montevideo str. IA_2009159199	assembly	103	4409	O:7	orange
**51979**	S.Montevideo str. IA_2010008282	assembly	81	4381	O:7	orange
**51981**	S.Montevideo str. IA_2010008283	assembly	165	4472	O:7	orange
**51983**	S.Montevideo str. IA_2010008284	assembly	123	4414	O:7	orange
**51985**	S.Montevideo str. IA_2010008285	assembly	42	4350	O:7	orange
**51989**	S.Montevideo str. IA_2010008287	assembly	70	4381	O:7	orange
**51381**	S.Montevideo str. MB101509-0077	assembly	52	4354	O:7	orange
**51383**	S.Montevideo str. MB102109-0047	assembly	185	4477	O:7	orange
**51385**	S.Montevideo str. MB110209-0055	assembly	194	4483	O:7	orange
**51387**	S.Montevideo str. MB111609-0052	assembly	32	4343	O:7	orange
**49405**	S.Montevideo str. MD_MDA09249507	assembly	44	4343	O:7	orange
**46903**	S.Montevideo str. NC_MB110209-0054	assembly	89	4408	O:7	orange

GPID	Organism	Status	Contigs	Genes	Serogroup (O antigen)	color
**46905**	S.Montevideo str. OH_2009072675	assembly	116	4452	O:7	orange
**20993**	S.Paratyphi C srt. RKS4594	complete	2	4690	O:7	orange
**30831**	S.Tennessee str. CDC07-0191	assembly	94	4546	O:7	orange
**20595**	S.Virchow str. SL491	assembly	5	4597	O:7	orange
**20593**	S.Hadar str. RI_05P066	assembly	50	4487	O:8	yellow
**20069**	S.Kentucky str. CDC 191	assembly	53	4383	O:8	yellow
**19457**	S.Kentucky str. CVM29188	assembly	4	4748	O:8	yellow
**20047**	S.Newport str. SL317	assembly	63	4720	O:8	yellow
**18747**	S.Newport str. SL254	complete	3	4710	O:8	yellow
**56085**	S.Dublin str. 3246	assembly	30	4717	O:9	red
**19467**	S.Dublin str. CT_02021853	complete	2	4684	O:9	red
**30687**	S.Enteritidis str. P125109	complete	1	4363	O:9	red
**30689**	S.Gallinarum str. 287/91	complete	1	4466	O:9	red
**56089**	S.Gallinarum str. 9	assembly	4	4579	O:9	red
**20049**	S.Javiana str. GA_MM04042433	assembly	19	4221	O:9	red
**236**	S.Typhi str. CT18	complete	3	5065	O:9	red
**371**	S.Typhi str. Ty2	complete	1	4632	O:9	red
**20591**	S.Weltevreden str. HI_N05-537	assembly	81	4784	O:9 or O:3,10	cyan
**13030**	S.arizonae serovar 62:z4,z23:-- str. RSK2980	complete	1	4278	O:3,10	gray

All Salmonella genomes were downloaded from NCBI except S. Typhimurium str. DT104, which downloaded from Sanger’s bacterial genome database.

All listed genomes are from subspecies *enterica* with the exception of the single *S. enterica subsp. arizonae* genome.
