# Supplementary figures and images for: Genomic variation in Salmonella enterica core genes for epidemiological typing
Source: BMC Genomics. 2012 Mar 12;13:88. doi: 10.1186/1471-2164-13-88 (PMC3359268; doi:10.1186/1471-2164-13-88)

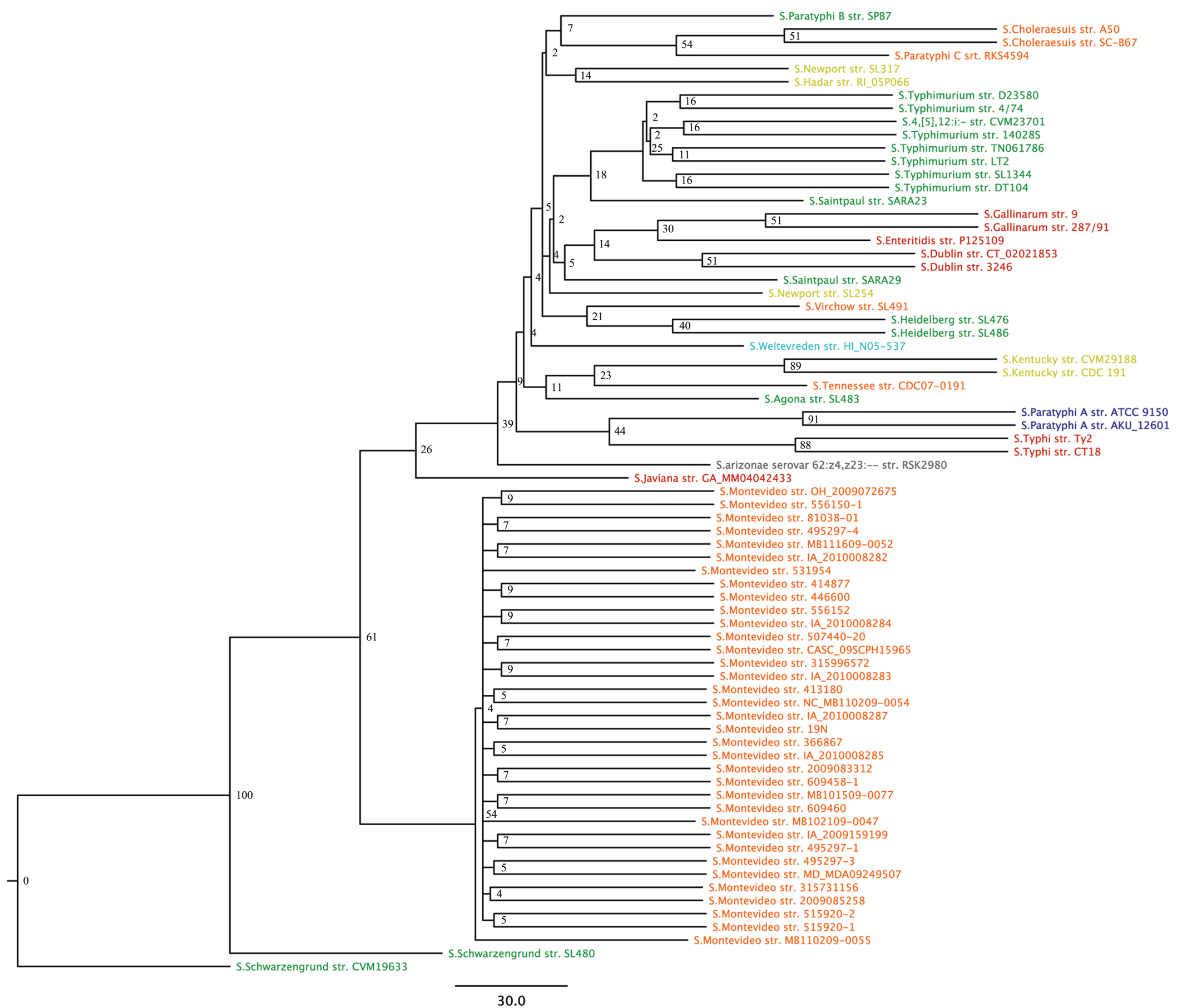

Supplement: Additional file 3 — Figure S1 Consensus tree based on 69 specific Salmonella genes. [file 1471-2164-13-88-S3.PDF]

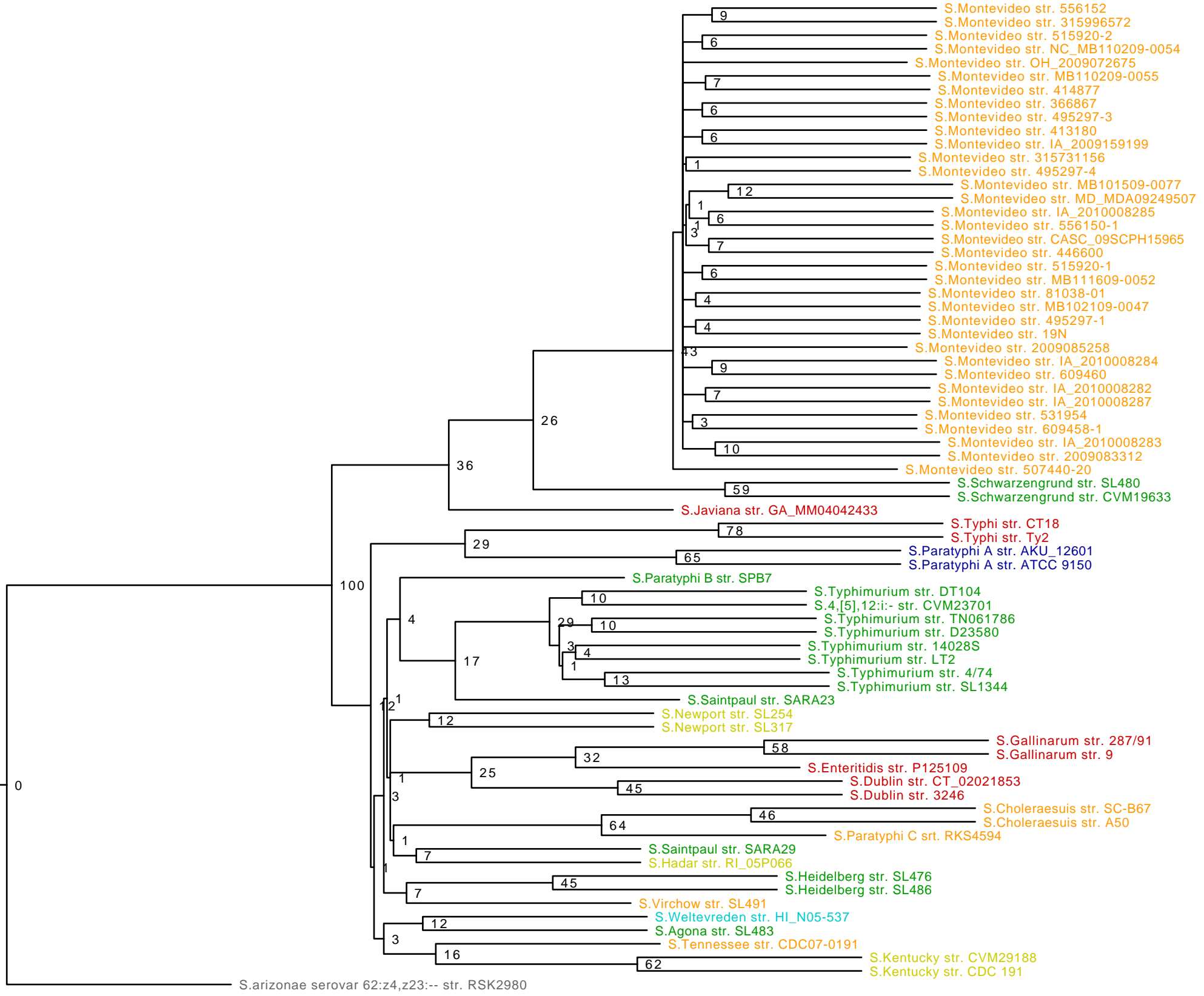

40.0

Supplement: Additional file 4 — Figure S2 Consensus tree based on 69 Salmonella core genes randomly picked up from high, medium and low variable core genes. [file 1471-2164-13-88-S4.PDF]

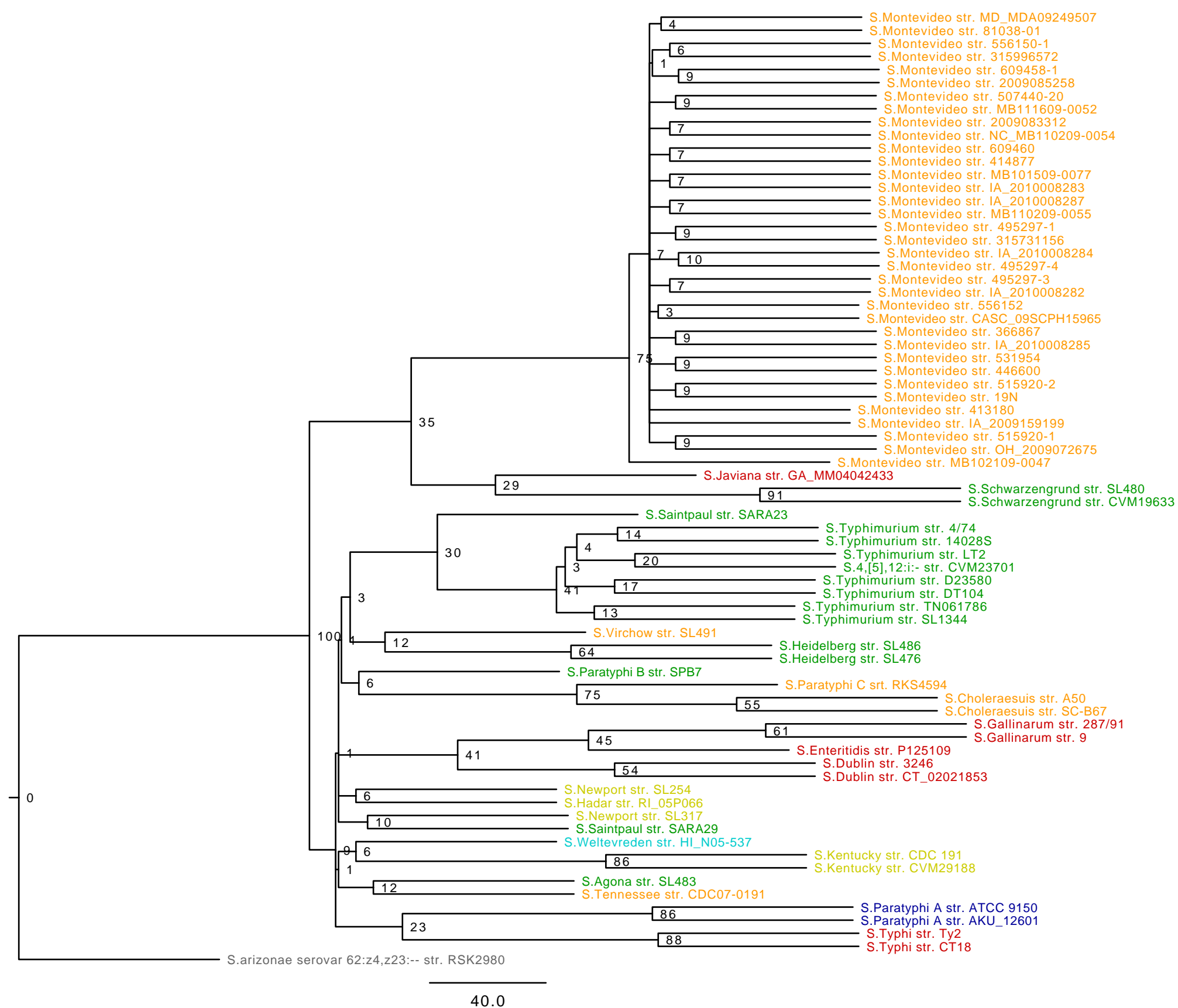

Supplement: Additional file 5 — Figure S3 Consensus tree based on 69 Salmonella core genes randomly picked up from medium variable core genes. [file 1471-2164-13-88-S5.PDF]

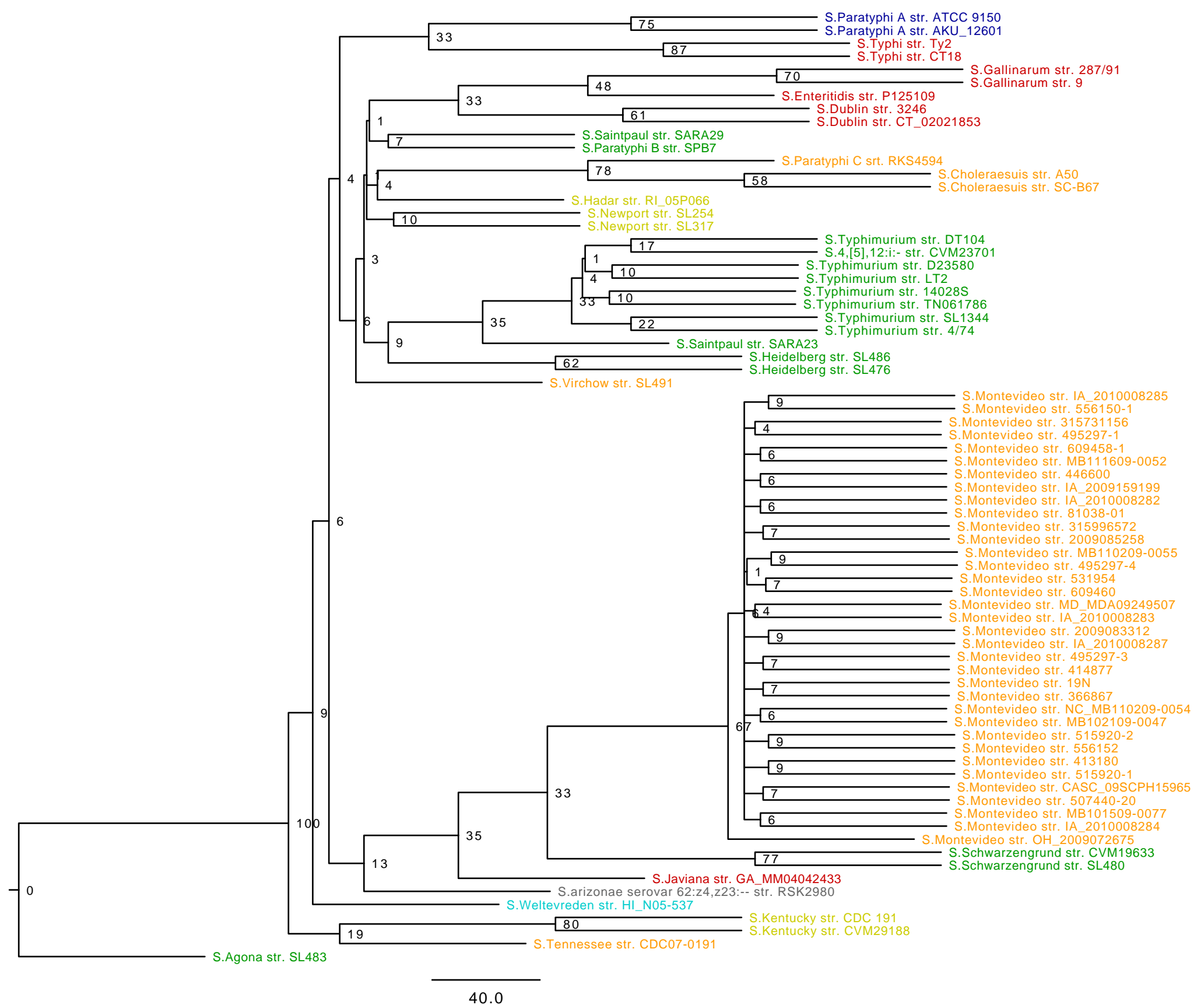

Supplement: Additional file 6 — Figure S4 Consensus tree based on 69 Salmonella core genes randomly picked up from highly variable core genes. [file 1471-2164-13-88-S6.PDF]

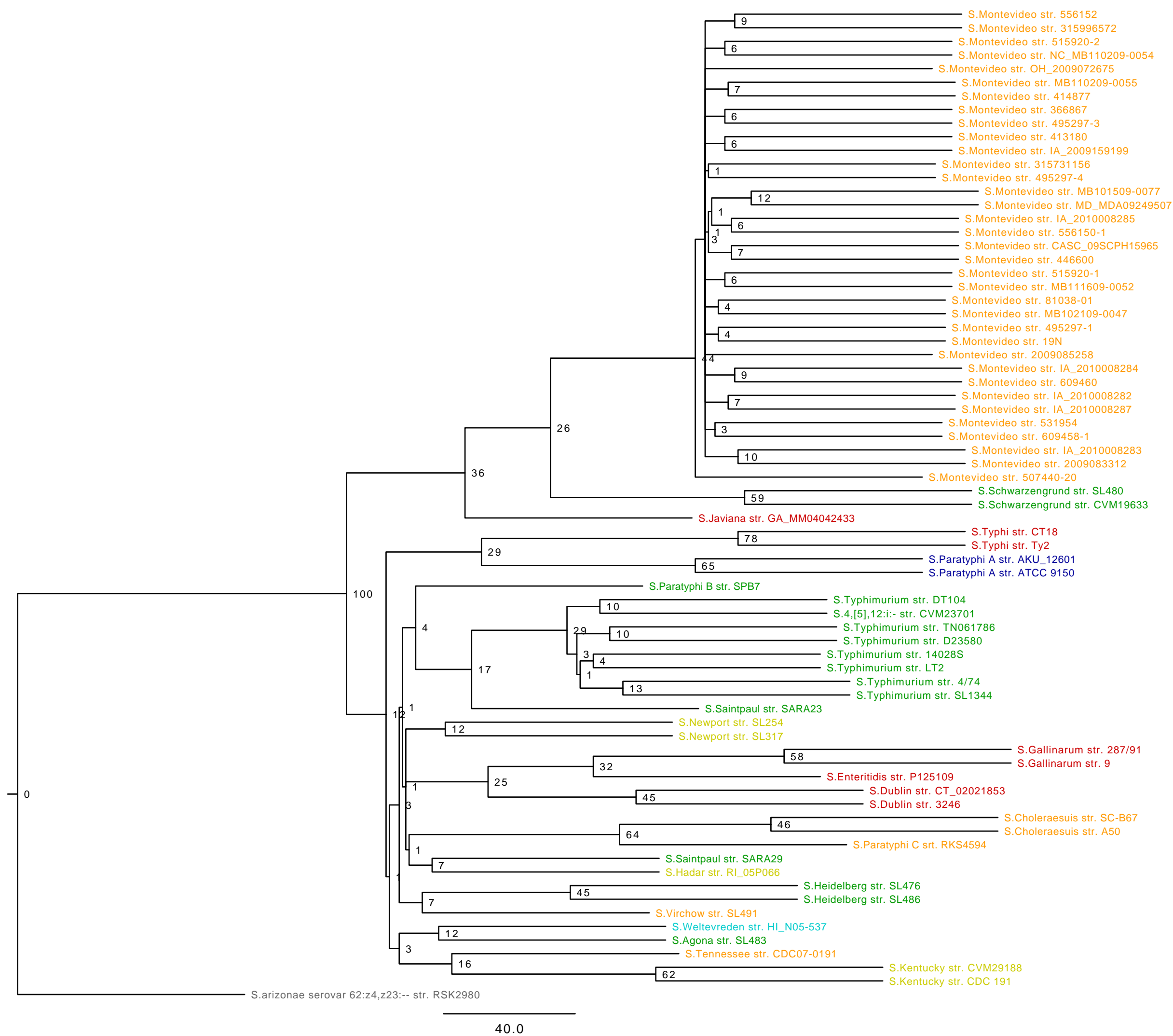

Supplement: Additional file 7 — Figure S5 Consensus tree based on 69 Salmonella core genes randomly picked up from decreasing curve in the variation plot. [file 1471-2164-13-88-S7.PDF]
